# Australian Wild Rice Populations: A Key Resource for Global Food Security

**DOI:** 10.3389/fpls.2019.01354

**Published:** 2019-10-22

**Authors:** Robert J. Henry

**Affiliations:** Queensland Alliance for Agriculture and Food Innovation, University of Queensland, Brisbane, QLD, Australia

**Keywords:** wild rice, *Oryza*, domestication, genetic diversity, Australia

## Abstract

Rice is one of the most important food crops contributing to the diet of large numbers of people especially in Asia. Rice (*Oryza sativa*) was domesticated in Asia many thousands of years ago and more recently independently in Africa. Wild rice populations are found around the tropical world. The extensive production of rice in many areas has displaced the wild populations that were the basis of the original domestications by humans. Recent research, reviewed here, has identified wild rice species in northern Australia that have been isolated from the impact of domestication in Asia. Wild rice populations contain novel alleles that are a source of desirable traits such as erect habit, disease resistance, large grain size, and unique starch properties. These populations include the most divergent genotypes within the primary gene pool of rice and more distant wild relatives. Genome sequencing also suggests the presence of populations that are close relatives of domesticated rice. Hybrid populations that demonstrate mechanisms of ongoing evolution of wild *Oryza* have been identified in the wild. These populations provide options for both new domestications and utilization of novel alleles to improve or adapt domesticated rice using conventional or preferably new breeding technologies. Climate change and growing food demands associated with population and economic growth are major challenges for agriculture including rice production. The availability of diverse genetic resources to support crop adaptation and new crop domestication is critical to continued production, and increased efforts to support *in situ* and *ex situ* conservation of wild *Oryza* and related species are warranted.

## Introduction

Rice is a key food crop with ongoing need for genetic improvement to satisfy food security. The *Oryza* genus is distributed around the tropical world. Domesticated rice has been cultivated in many of the areas that would have been native habits for wild *Oryza* species. Australia is a region that has escaped from the impact of rice domestication until very recently resulting in the persistence of many extensive populations of wild *Oryza* (Henry et al., 2010) across a very large area of northern Australia.

Advances in DNA sequencing technology have made it possible to analyze the genomes of large numbers of individuals. This has had significant impact on human genetics but also on rice biology revealing much about the diversity of the populations, the relationships between different populations, and the evolutionary events leading up to domestication by humans (Wambugu et al., 2018).

### *Oryza* Genus

**Figure 1:**
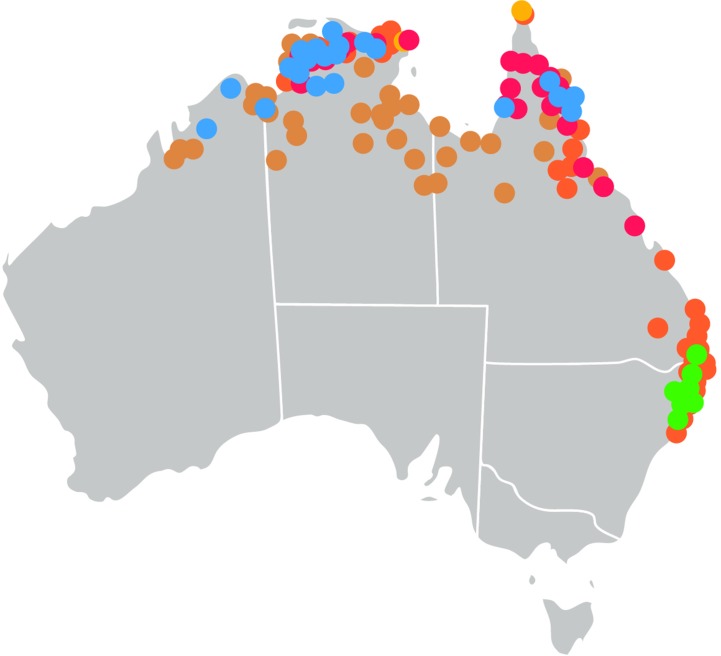
Distribution of wild relatives of rice in Australia. Locations of collections of wild relatives of rice in Australia as detailed in the flora of Australia *(Flora of Australia* Volume 44A *Poaceae* 2, Melbourne: ABRS/CSIRO 2009). (Red) *Oryza rufipogon*, (Blue) *Oryza meridionalis*, (Yellow) *Oryza officinalis*, (Brown) *Oryza australiensis*, (Green) *Potamophila parviflora*, (Orange) *Leersia hexandra*.

The *Oryza* genus (Mondal and Henry, 2018) is pantropical in distribution with two domesticated and 24 wild species. Rice was domesticated in Asia as *O. sativa* and in Africa as *Oryza glaberrima*. The AA genome group (*O. sativa* complex) includes the two domesticated species and six wild relatives. The genus has 11 genome types with the AA, CC, and EE genomes types found in Australia. Other members of the *Oryzeae* tribe are also found in Australia (**Table 1**). These taxa are found in northern and eastern Australia (**Figure 1**).

**Table 1:** The *Oryzeae* tribe in Australia.

Species	Genome	Notes
*Oryza meridionalis*	AA	Found in Australia and extends to New Guinea
*Oryza rufipogon*	AA	Populations in Australia have a chloroplast genome closer to *O. meridionalis* than *O. rufipogon* from Asia and may be best considered as a distinct taxa.
*Oryza officinalis*	CC	Reported from only a few locations
*Oryza australiensis*	EE	Endemic and widespread in northern Australia
*Potamophila parviflora*	*not Oryza*	Found only in a few rivers on the central east coast
*Leersia hexandra*	*not Oryza*	Perennial from eastern and northern Australia

### AA Genome Populations in Australia

The close relatives of domesticated rice, the AA genome species, represent the primary gene pool of rice. The close relatives of domesticated rice in northern Australia have been shown to be highly diverse, including at least two distinct taxa and represent a yet unexplored wider genetic resource for global rice production. Populations have been identified with morphological and genetic characteristics suggesting a hybrid origin and some degree of ongoing gene flow between these taxa that could support continued rapid evolution of the wild *Oryza* in Australia. These Australian *Oryza* could be critical in adaptation of rice to rapid climate change and changing consumer demands and preferences. Recent evidence also suggests that, despite rice being domesticated in Asia (Huang et al., 2012), the Australian populations have contributed genes to domesticated rice (Fujino et al., 2019). The AA genomes species in Australia have been generally classified as *Oryza meridionalis* or *Oryza rufipogon*.

#### Oryza meridionalis

**Figure 2:**
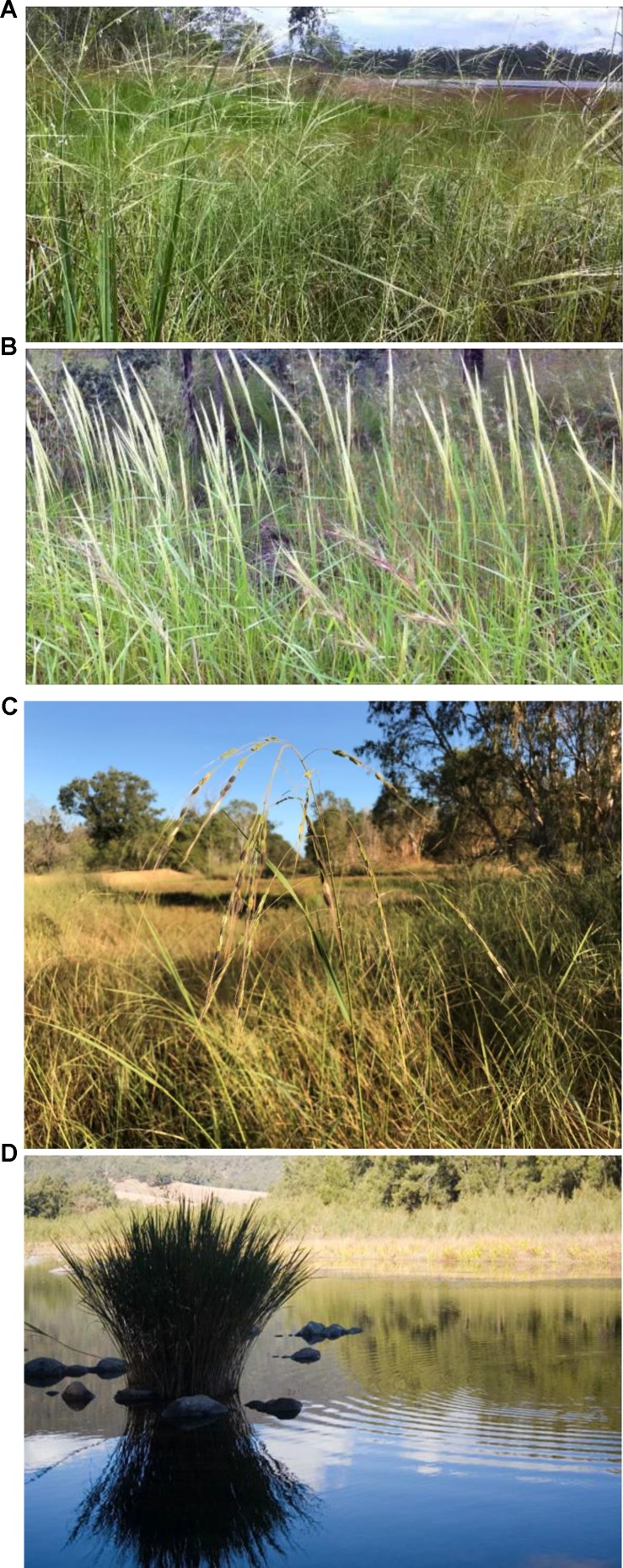
Australian wild rice **(A)** *Oryza.rufipogon*, **(B)** *O. meridionalis*, **(C)** *O. australiensis*, and **(D)** *Potamophila parviflora*.

*Oryza meridionalis* (**Figure 2**) was first described relatively recently, in 1981, being most readily distinguished from *O. rufipogon* in the field by having smaller anthers, longer awns, and closed panicles. This species had been considered to be an annual, but more recent research has identified widespread populations of perennials in northern Queensland (Sotowa et al., 2013). The distinctness of the annual and perennial types has not been established, but both types are evidenced by the survival or otherwise, of plants from different populations, in the glasshouse after flowering (Sotowa et al., 2013).

#### Oryza rufipogon

*O. rufipogon* (**Figure 2**) was described by Griff (1851). Earlier taxonomy had included these plants in the wider group known as *Oryza perennis*. The Australian populations show significant molecular differences from Asian *O. rufipogon* and share some chloroplast sequence homology with *O. meridionalis* (Waters et al., 2012; Brozynska et al., 2014) suggesting that they might best be considered as distinct taxa. The morphological differences between Asian and Australian *O. rufipogon* are not clear with limited data from common garden experiments that would allow direct analysis of quantitative traits in the same environment. *O. rufipogon* in Australia can be most easily distinguished from *O. meridionalis* in the field by the presence of open panicle, shorter awns, and larger anthers. *O. rufipogon* and *O. meridionalis* may be confused in some Australian herbarium records with many collections being prior to the publication of *O. meridionalis*. This is further complicated by the potential for rare hybrid populations. The hybrid population has mixtures of the distinguishing traits described above. An analysis of the current distribution of these taxa and their hybrids would require sampling over a very wide area in Queensland, Northern Territory, and Western Australia.

### Molecular Analysis of Australian AA Genome Species

#### Chloroplast Genomes

The sequence of the chloroplast genome has been widely used to determine evolutionary relationships in plants (Wambugu et al., 2015). The advantages of this approach include the highly conserved nature of the chloroplast making direct comparisons of the same sequence in plants less difficult and providing a significant amount of DNA sequence to compare. Comparison of the chloroplast sequences of wild rices that are closely related to domesticated rice has defined events leading up to rice domestication.

Analysis of the chloroplasts of Australian AA genome (Nock et al., 2011) indicated that chloroplast genomes are distinct from those of other populations in Asia (Waters et al., 2012; Brozynska et al., 2014). These species have been used to develop methods for the assembly of an accurate complete chloroplast genome sequence from short read sequence data (Nock et al., 2011). These methods have used mapping to a close reference and *de novo* assembly and rationalized the differences obtained by these two approaches (Guyeux et al., 2019).

Analysis of Australian AA genome populations confirmed the presence of two distinct chloroplast genomes that form an Australian clade that is an outgroup relative to all Asian and African populations (Moner et al., 2018). A survey of the whole chloroplast genomes of 58 genotypes of wild rice showed that the distinct Australian clade extended north to the Philippines (Moner et al., 2018). The study also confirmed the close relationships between the chloroplast sequences of *Oryza glumipatula* in South America and *Oryza longistaminata* in South Africa. This suggests inter-continental exchange of genetic material relatively recently. This exchange can be viewed as part of ongoing movement of wild rice around the tropics with birds as likely vectors. The presence of a wild rice in the Philippines with an Australian chloroplast type may be further evidence of long distance dispersal events.

Modern domesticated rices can be divided into several types including *indica*, *japonica*, and *aus* types that may have resulted from separate domestication events (Civan et al., 2015). The *indica* and *japonica* types correspond to distinct chloroplast sequences in wild populations indicating capture of these in separate domestication events. The *aus* type was found to include individuals with either wild chloroplast types (Moner et al., 2018) indicating a more complex genetic origin (Choi et al., 2017).

#### Nuclear Genomes

Around 3 million years ago, a divergence in the wild rice populations that are progenitors of domesticated rice and still inter-fertile with domesticated rice resulted in a lineage that became *O. meridionalis* (Moner and Henry, 2018), a species found in Australia, separating from those leading to *O. rufipogon*, the wild rice found in Asia. Analysis of the nuclear genome shows that the Australian populations of that have been considered to be *O. rufipogon* diverged from those in Asia around 1.7 million years ago (Brozynska et al., 2017). The available evidence suggests that these *O. rufipogon* like Australian populations should also be considered as a separate species. The divergence of the Asian and African wild rices in the primary gene pool of rice was shown to be more recent.

The genome of the *O. rufipogon* like Australian wild rice showed some evidence for possible introgression of chromosomal regions from wild *indica* types (Brozynska et al., 2017).

Populations with a mixture of the traits of different taxa have been identified in the wild in Australia (Moner et al., 2018). These populations have been confirmed as hybrids by examination of the chloroplast and nuclear genomes. Plant populations were identified with characteristics intermediate between those of the two AA genome taxa. The panicle varied from opened to closed and the anther length varied. The nuclear genome was divergent and, despite morphology similar to *O. meridionalis*, the chloroplast was that of the *O. rufipogon* type. Field collections of 29 populations (Moner et al., 2018) from northern Queensland have been made from the northern to the southern extremes of the current natural distribution. These collections are the subject of ongoing analysis by DNA sequencing.

Comparison with the genome of domesticated rice suggest that the Australian wild species have significant diversity, even in chromosomal regions of very low diversity (“genome deserts”) in the domesticated rice gene pool (Krishnan et al., 2014), making them import sources of novel diversity for rice improvement. These regions of low diversity suggest that selective sweeps may have occurred in wild populations, possibly due to pests or diseases, and that these areas of the genome may have been further depleted of variation by bottlenecks during domestication.

### Other Related Species

Other more distant relatives of rice are also found in the Australian flora. They have generally received less research attention than the Australian members of the AA genome clade. These more divergent species include *Oryza* species, *Oryza officinalis*, and *Oryza australiensis* and other species from within the tribe *Oryzeae* such as *Potamophila* (Abedinia et al., 1998) and Leersia and other species from the subfamily *Ehrhartoideae* such as *Microlaena stipoides* (Shapter et al., 2013). These more diverse species also have genes that may be useful in rice improvement with more difficulty. However, despite it being a more distant relative of rice, *O. australiensis* has already been used successful as a genetic resource in rice improvement. Techniques such as gene editing may allow more rapid deployment of useful novel alleles, from all of these wild relatives, in the domesticated gene pool.

#### Oryza officinalis

*O. officinalis* (CC genome) has been reported only in the extreme north of Australia on the mainland and on Moa Island in the Torres Strait. Collect of this species has not been reported recently, and the status of the Australian populations is uncertain. The author was unable to locate populations on Moa Island during a visit in 2016. This species is part of the CC genome group of *Oryza* species. Populations reported in Australia may not be permanently established and may be the result of occasional migration from Asia suggesting that the current presence of this species in Australia requires confirmation.

#### Oryza australiensis

*O. australiensis* (EE genome; **Figure 2**) is widespread across northern Australia. This species is usually perennial and grows in seasonally dry environments as it can survive the dry season as a rhizome. This species is often found in areas surrounding permanent water holes or lakes that support perennial AA genome *Oryza* or temporary bodies of water in depressions supporting annual AA genome *Oryza* in the wet season but also extending further away from the surface water on waterlogged soils beyond these water bodies. The plants can be very tall reaching 3 m in height but can also be observed as very small plants in environments that limit growth such as populations growing on subsoil in roadside drains (personal observations).

#### Potamophila parviflora

River grass (*Potamophila parviflora*; **Figure 2**) is found in just a few rivers (Manning, Hastings, Clarence, and Richmond Rivers) on the central east coast of Australia in northern New South Wales (Abedinia et al., 1998). It appears to grow only in the shallow running water of these rivers. This monotypic genus is probably more closely related to *Zizania* of North America than to *Oryza*. Cold adaptation and the presence of separate male and female flowers are potentially useful traits. This species grows just south of the border between Queensland and New South Wales but has not been reported in Queensland despite some searching by the author and others.

#### Leersia hexandra

*Leersia hexandra* is a perennial found in northern and eastern Australia from the Northern Territory to north-eastern New South Wales. The use of species of *Leersia* as food has not been documented. Plants have recently been collected in urban areas close to Brisbane. The exotic *Leersia oryzoides* is also found in Australia.

#### Microlaena stipoides

Weeping grass (*M. stipoides*) is found throughout temperate Australia demonstrating adaptation to cooler climates. Attempts have been made to domesticate this species as a cool climate dryland rice crop. Mutagenesis (Shapter et al., 2013; Malory et al., 2011) has allowed recovery of non-shattering types and this together with selection for large grain has generated genotypes with potential for grain production. This widespread species is often found in shaded environments and has been used as a lawn grass.

### Useful Traits for Rice Improvement in Wild Australian Populations

The capture of genes from *O. meridionalis* has been successful because, although the F1 hybrids with *O. sativa* have pollen sterility, they retain female fertility allowing backcrossing of desirable traits into domesticated rice (Yoshimura et al., 2010). Potentially useful traits include grain yield, grain size, grain color, starch properties, cooking qualities, eating qualities, and disease resistances. The genes responsible for these traits have not been characterized to date, but the availability of genome sequence data and more intensive collections of the populations of these taxa will facilitate the identification of useful genes and alleles.

#### Yield

The yield of Australian wild rices in cultivation has not been evaluated. The difficulty of cultivating a species that is so prone to shattering has made this difficult. The abundant grain observed on plants in the wild suggests that good yields might be achieved if this problem was overcome. The large grain size of some Australian wild rices may be a trait that could contribute to yield. Yield evaluation in comparative trials has not been conducted for any of these species.

#### Grain Quality

The grains of Australian wild rice vary from medium to long grain and include some with large grain size (Tikapunya et al., 2017). Transfer of this trait to domesticated rice is in progress. The grains of the A genome species are long or medium while *O. australiensis* grains are short. The wild rice grains are all slender when compared to domesticated rice. The impact of nutrition on grain filling and grain size has not been examined for these species, and improved grin size may be possible with optimal plant nutrition.

Australian wild rice has a color that would be described as red in rice color language. The Australian populations have colors that appear to be distinct from that of Asian red rices that have been available for comparisons. This suggests that a key market option for these species is as colored rice that has not been milled.

The starch properties of rice reflect the eating and nutritional qualities of the grain (Wang et al., 2015). The Australian species have unique starch properties (Kasem et al., 2012–Tikapunya et al., 2017). All taxa (including AA genome species and *O. australiensis*) had high amylose contents and gelatinization temperatures. Pasting properties varied. Analysis of starch structure suggested the presence of shorter chain amylose in *O. australiensis* (Tikapunya et al., 2017). Much remains to be understood about the novel starch properties of these rice species and the extent of variation in wild populations. The genetic basis of the starch properties is also not determined.

Sensory evaluation of Australian wild rice has indicated acceptable eating qualities (Tikapunya et al., 2018). Australian wild rices have been compared with Asian domesticated rices (including long grain, medium grain, basmati, red basmati, and red rice) and with Canadian wild rice (*Zizania palustris*). Specific descriptors were developed for sensory evaluation of these rices. The Australian wild rice was similar to the red rice and red basmati having a mild aroma and flavor but without the lingering aftertaste. The wild rice had a firmer texture and required a longer cooking time. The overall cooking profiles, sensory, and physical attributes suggest and that Australian wild rice has potential for commercialization in the colored rice market and may be a useful genetic resource for rice breeding.

#### Disease Resistance

Production of domesticated Asian rice in northern Australia has been limited by a high incidence of disease relative to rice production further south in Australia. The southern production areas have been well outside the natural range of rice and rice pathogens. Rice and associated rice pathogens (Khemmuk et al., 2016) are native to northern Australia and a wide range of pathogen species are present. Much of the diversity of these organisms remains to be explored. Native Australian rices have resistance to the local strains of rice pathogens and should provide a useful source of resistance to local diseases for use in breeding rice varieties for this region. Asian domesticated rice varieties have not been developed to have resistance to the native rice pathogens of northern Australia. The wild populations are key resources for the successful establishment of rice production on the large areas of land that are suitable for production in northern Australia. Crops of domesticated rice have been devastated by disease in Northern Australia while surrounded by wild populations that appear resistant.

### Human Impact on Populations

The wild populations are extensive in the extreme north but are threatened by developments including the improvements of roads and the resulting more rapid spread of invasive weeds into new areas, associated with increased traffic. Aquatic weeds are displacing wild populations at some locations including protected areas such as national parks. Greater awareness of the presence of these populations and their potential to contribute to global food security is necessary to ensure adequate measures are taken to protect the wild populations.

The Australian landscapes in which rice is found in Australia have been inhabited by humans for a very long time. The way in which rice was used as food is not well documented. It is possible that human use has impacted on the genetics of wild rice populations in Australia. The large grain size of some Australian populations (Sotowa et al., 2013) might reflect some human selection. Shattering is a key trait associated with domestication of grains. The wild species are all extremely prone to shattering suggesting little progress toward a non-shattering domesticated type. However, recent evidence shows that domesticated populations can rapidly revert to shattering types under natural selection (Zeng et al., 2018).

### Potential for New Domestications

In addition to their use in rice improvement, the Australian *Oryza* could be targeted for direct domestication as new rice types. The key trait to be overcome in domestication is probably shattering, with wild populations all demonstrating high shattering characteristics. The option of domestication needs to be considered along with the potential to source many genes for useful traits in these populations for transfer into domesticated rice. The traits that are most desirable are disease resistance, grain size, grain color, and grain quality (starch properties). The strategy of new domestication may be especially useful in developing rice varieties adapted to new climates with advancing or rapid climate change especially with the availability of extensive genomics resources for rice (Abberton et al., 2016).

## Conclusions

Wild rice populations in Australia include species at varying distances from domesticated rice. These provide a rich resource for use in rice improvement. Both novel alleles and novel traits might be sourced from the Australian wild genotypes. Increased efforts to conserve these genetic resources would be justified. The current very limited *ex situ* collections in seed banks need to be supplemented by much more extensive collections. The *in situ* conservation of these species would be aided by greater awareness of the populations and their importance and may involve efforts to control weeds invading the aquatic habitats of the wild *Oryza*. Revision of the taxonomy of the A genome species is suggested by the available molecular evidence. Clarification of the taxonomy may aid conservation efforts by ensuring efforts to conserve rarer populations, including suspected hybrid populations. The growing genomic resources for the *Oryza* genus (Stein et al., 2018) would be extended usefully by more efforts on the relatively poorly characterized Australian members of this group especially as they include the more divergent members of the A genome clade.

## Author Contributions

This manuscript is the work of the author.

## Funding

The Australian Research Council provided support for much of the research reviewed here.

## Conflict of Interest

The author declares that the research was conducted in the absence of any commercial or financial relationships that could be construed as a potential conflict of interest.
